# Quantitative evaluation of intraorbital optic nerve in optic atrophy using diffusion tensor imaging

**DOI:** 10.1038/s41598-022-16267-3

**Published:** 2022-07-15

**Authors:** Eun Hee Hong, Jin-Ju Yang, Yeji Yeon, Hyun Soo Cho, Ji Young Lee, Won June Lee, Yu Jeong Kim, Yeji Moon, Han Woong Lim

**Affiliations:** 1 Department of Ophthalmology, Hanyang University Hospital, Hanyang University College of Medicine, 222-1, Wangsimni-ro Seongdong-gu, Seoul, 04763 South Korea; 2 Hanyang Vision Research Center, Hanyang University, Seoul, South Korea; 3 Department of Ophthalmology, Hanyang University Guri Hospital, Guri, South Korea; 4 Department of Radiology, Seoul St. Mary’s Hospital, Seoul, South Korea; 5 Department of Ophthalmology, Asan Medical Center, University of Ulsan College of Medicine, Seoul, South Korea

**Keywords:** Optic nerve diseases, Image processing, Diseases of the nervous system, Visual system, Medical research, Magnetic resonance imaging

## Abstract

The aim of this study is to quantitatively investigate the microstructural properties of the optic nerve (ON) in vivo using diffusion tensor imaging (DTI) in patients with unilateral optic atrophy (OA) and to determine their association with retinal nerve fiber layer (RNFL) thickness of the optic nerve head (ONH). Six patients with unilateral OA and 11 control subjects underwent DTI. ONs from ONH to the orbital apex were tracked. Fractional anisotropy (FA), mean diffusivity (MD), axial diffusivity (AD), and radial diffusivity (RD) were computed in both ONs and their correlation with RNFL thickness measured using optical coherence tomography was also analyzed. FA of atrophic ON was lower than that of non-affected and control ONs (atrophic [A], 0.136 ± 0.059; non-affected [N], 0.384 ± 0.048; control [C], 0.389 ± 0.053). MD and RD of atrophic ONs were higher than those of non-affected and control ONs (MD, A, 0.988 ± 0.247; N, 0.658 ± 0.058; C, 0.687 ± 0.079; RD, A, 0.920 ± 0.247; N, 0.510 ± 0.054; C, 0.532 ± 0.078). All DTI measures of atrophic ON except for AD showed a significant correlation with RNFL thickness of ONH; FA showed the strongest correlation, followed by RD and MD (FA, *R*^2^ = 0.936, *P* < 0.001; RD, *R*^2^ = 0.795, *P* < 0.001; MD, *R*^2^ = 0.655, *P* = 0.001). This study reports quantitative analysis of the ON using DTI and differences in DTI measures between atrophic and normal ONs. The significant correlation between DTI measures and RNFL thickness suggests the applicability of DTI as a clinical tool to evaluate the ON.

## Introduction

The optic nerve (ON) consists of bundles of retinal ganglion cell (RGC) axons, which transmit visual stimulus from the RGC to the lateral geniculate nucleus, where most of the neuron’s synapse. The ON is the only cranial nerve that leaves the cranial cavity and is considered part of the central nervous system (CNS)^1^. RGC axons comprise the retinal nerve fiber layer (RNFL) in the retina, converge at the optic disc (optic nerve head) and leave the eye to form the ON^1^. Detecting changes in the RNFL thickness can be achieved with optical coherence tomography (OCT), which objectively analyzes each layer of the retina. Currently, the use of OCT to measure peripapillary and macular RNFL thickness is considered the most useful clinical examination in ON diseases. Magnetic resonance imaging (MRI) or visual evoked potential (VEP) can also be used to evaluate the structural or functional loss of the ON. However, in conventional MRI, only structural changes of the ON can be observed, and in VEP, only the approximate loss of function of the ON can be assessed.

Diffusion tensor imaging (DTI) is one of the MRI techniques based on diffusion MRI (dMRI) that generates images through mapping the relative diffusion process of water molecules in the biological tissues^2^. DTI relates image intensity to the relative mobility and direction of water molecules within the tissue, noninvasively providing information regarding the fiber architectures of the brain white matter^3^. It has been well established for mapping brain microstructure and white matter tracts in vivo. It is a useful non-invasive approach to obtain information about the microscopic physical properties of tissues and assess anatomical connectivity in the human brain^4–6^. Most dMRI studies rely on DTI-derived indices such as fractional anisotropy (FA) and mean diffusivity (MD, μm^2^/s), which represent overall diffusion properties. FA describes a normalized measure of the uniformity of diffusion of water molecules with larger values indicating greater anisometry and represents the overall microstructural integrity^7,8^. MD describes the magnitude of diffusion according to the direction relative to the fiber tracts and may reflect more specific pathophysiological changes such as axonal and myelin integrity^7,8^. The diffusivity can also be measured more precisely as axial diffusivity (AD, μm^2^/s) and radial diffusivity (RD, μm^2^/s), representing diffusion parallel to and perpendicular to the long axis of specific white matter tracts, respectively^9,10^.

Changes in the neural structure or microstructural properties of the ON can hardly be detected using the commonly used protocols of MRI. In ON fibers which consist of bundles of neuronal axons, a significant part of these structures are coherently oriented along with the directions of the fibers, resulting in a macroscopic orientation dependence of the measured diffusion-weighted MR signal. Therefore, the diffusion properties of ON can be regarded to represent various neural states and enable quantitative evaluation of ON in various pathologic conditions. Several studies of ON disease such as glaucoma, optic neuritis, and other optic neuropathies have used some DTI measures^11–14^. We aimed to evaluate the microstructural properties of in vivo ON using DTI by comparing DTI measures of normal and atrophic ONs, which is considered the most reliable and best way to identify the difference. To the best of our knowledge, the properties of ON in patients with optic atrophy (OA) have not been studied previously. We hypothesized that there would be a difference in DTI measures between atrophic and healthy optic nerves and that DTI measures would be related to OCT measurements in atrophic ONs. Therefore, in this study, we quantitatively investigated in vivo DTI measures of ONs in patients with unilateral OA and compared them to the contralateral normal ONs and ONs of normal subjects to identify the microstructural properties of the ON. Furthermore, we analyzed the association between DTI measures of the ON and RNFL thickness measured using OCT to evaluate the clinical utility of DTI measures.

## Methods

The study protocol was approved by the Institutional Review Board of Hanyang University Hospital (HYUH 2020-04-016). The study design followed the tenets of the Declaration of Helsinki for biomedical research. Written informed consent was obtained from all participants.

### Participants

Patients with unilateral OA (OA group) and age-matched control subjects (control group) with no eye disease were enrolled in this cross-sectional study between April 2020 and September 2021. OA group included six patients, and the control group included 11 subjects. All participants visited the Department of Ophthalmology at the Hanyang University Medical Center and underwent a complete ophthalmologic examination, including visual acuity testing, manifest refraction assessment, slit-lamp examination, intraocular pressure measurements, and swept-source OCT (SS-OCT; DRI-OCT Triton; Topcon, Tokyo, Japan). The SS-OCT wide scan protocol, which covers a 12 × 9 mm area of the posterior pole, was used, and RNFL thickness of optic nerve head (ONH) was measured by automated placement of a circle 3.4 mm in diameter, centered on the disc.

The inclusion criteria for patients with unilateral OA were as follows: unilateral OA (atrophic eye) and normal contralateral eye (non-affected eye); best-corrected visual acuity of 20/40 or better in the non-affected eye; no acute visual symptoms present; and the diagnosis was confirmed by two neuro-ophthalmology specialists (EHH and HWL). The exclusion criteria for patients with unilateral OA were as follows: history of ophthalmic surgery or any other ocular disease that could interfere with the visual function in the non-affected eye; history of neurological or psychiatric disease; spherical equivalent refractive errors of <  − 6.0D or >  + 6.0D in either eye; any media opacity that would significantly interfere with the acquisition of OCT images in either eye; and inability to perform OCT or MRI. The inclusion criteria for control subjects were as follows: no ocular disease that could interfere with the visual function in either eye; and no history of ophthalmic surgery in either eye. The exclusion criteria for control subjects were as follows: history of neurological or psychiatric disease; spherical equivalent refractive errors of <  − 6.0D or >  + 6.0D in either eye; any media opacity that would significantly interfere with acquisition of OCT images in either eye; and inability to perform OCT or MRI.

### MRI acquisition

Whole-brain DTI and T1-weighted scans were acquired on a 3T MRI scanner (Philips, Archive, Netherlands) with a 32-channel head coil. The following DTI parameters for two phase-encoding directions (anteroposterior and posteroanterior) with non-diffusion-weighted images (b = 0 s/mm^2^), and 48 diffusion gradient directions with b = 2000 s/mm^2^: echo-time/repetition time (TE/TR) = 85/5500 ms, 66 axial slices, slice thickness = 2 mm, voxel size = 2 mm isotropic, field of view (FoV) = 210 × 210 mm. For T1-weighted MRIs, the image parameters were as follows: TR = 4.6 ms; TE = 8.0 ms; flip angle = 8; matrix size = 224 × 224, 156 axial slices, slice thickness = 1 mm; voxel size = 0.7 × 0.7 × 1. The entire length and thickness of both ONs were identified or confirmed in each subject. The participants were asked to close their eyelids and reduce eye movements during the scan as much as possible.

### DTI preprocessing

**Figure 1:**
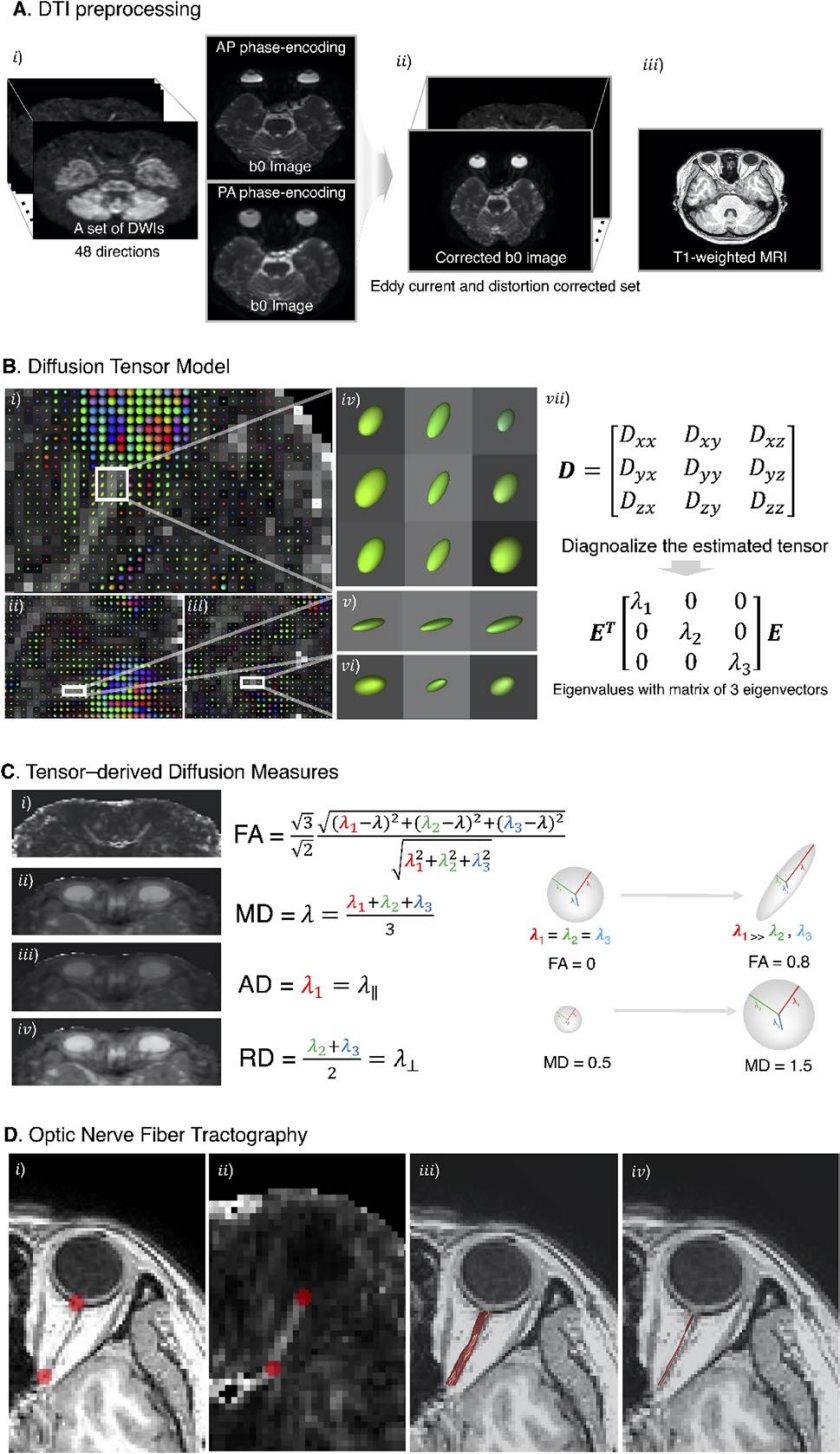
Overview of diffusion tensor imaging (DWI) analysis. (**A**) Preprocessing of raw DWI data. A set of 50 images was acquired from each participant, including 48 sampling directions and two b0 images of anteroposterior (AP) and posteroanterior (PA) phase-encoding directions (*i*). One corrected b0 image was generated, and the entire diffusion data was applied by estimating artifacts from two pairs of b0 images (*ii*). Finally, a set of 49 images was co-registered to the T1 image (*iii*). (**B**) Diffusion tensor model. To represent the optic nerve in the axial (*i*), sagittal (*ii*), and coronal (*iii*) views on the FA image, diffusion directionality was color-coded with green representing anterior–posterior directions, red representing left–right directions, and blue representing superior-inferior directions for a single subject using FSL eyes. A close-up view of 3 × 3 voxels of the optic nerve behind the eye globe in the axial (*iv*), three voxels in the sagittal (*v*), and three voxels in the coronal views (*vi*). The diffusion tensor D was estimated from a set of DWIs. The three orthotropic axes of a diffusion ellipsoid can be determined by the decomposition of the tensor into eigenvectors and eigenvalues (*vii*). (**C**) Tensor-derived diffusion measures. Cropped image of the fractional anisotropy (FA) (*i*), mean diffusivity (MD) (*ii*), axial diffusivity (AD) (*iii*), and radial diffusivity (RD) (*iv*). FA values are bounded between zero (a perfect sphere) and one (an infinitely long cigar shape). MD is the mean of the eigenvalues of the diffusion tensor. AD indicates an eigenvalue of λ_1_, which is diffusivity along the principal axes, while RD is diffusivity along non-principal axes by averaging the eigenvalues of λ_2_ and λ_3_. (**D**) Optic nerve fiber tractography. Two spherical regions of interest (ROIs) are placed posterior to the globe of the eye and in the orbital apex at the center of the annulus of Zinn to identify the optic nerve pathway. Placement of the two ROIs overlaid on T1 (*i*) and FA image (*ii*). Visualization for cleaned optic nerve tractography (*iii*) and a central fiber in the optic nerve passing through the ROI (*iv*).

**Figure 2:**
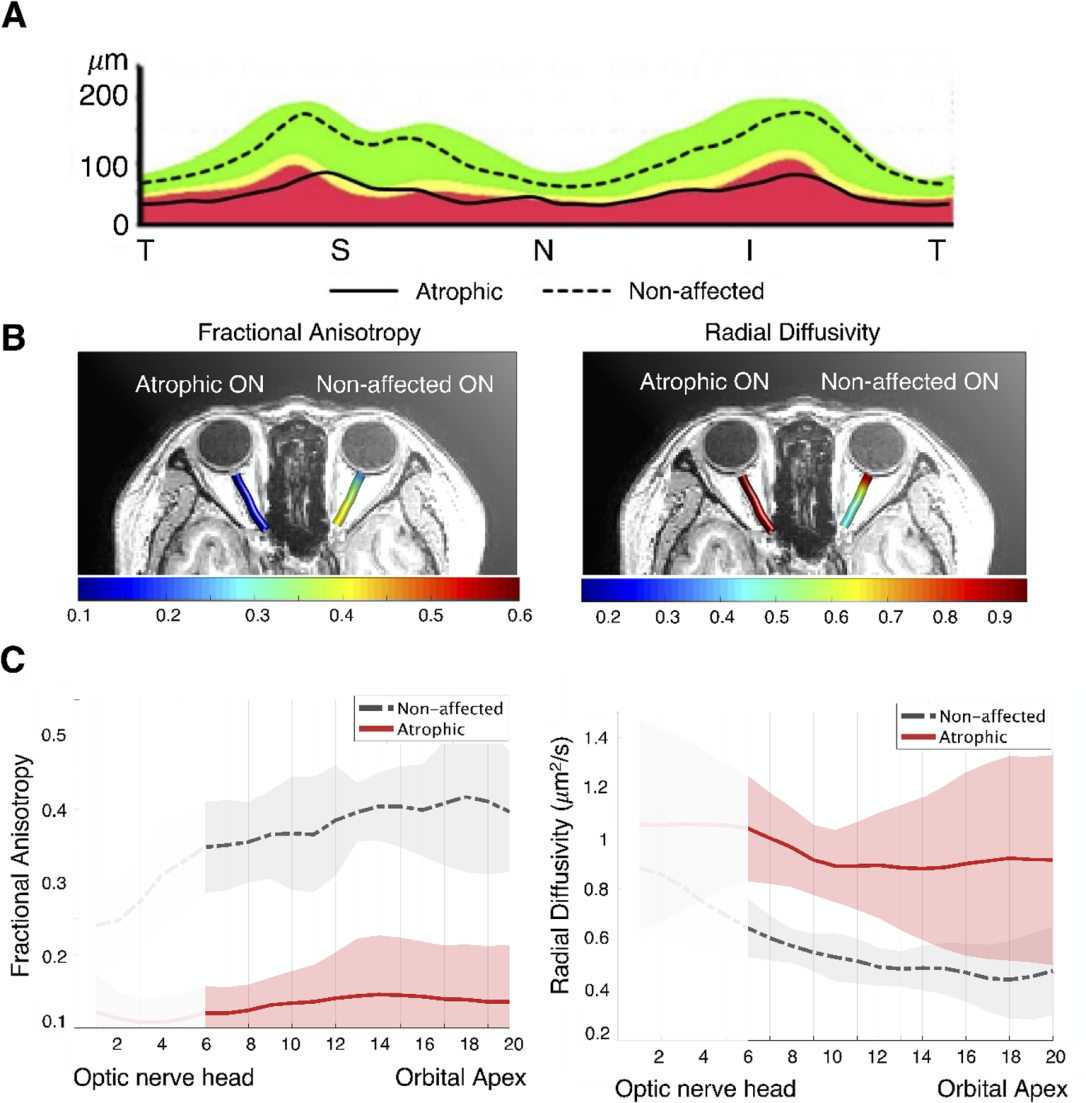
Visualization of the average peripapillary retinal nerve fiber layer (RNFL) thickness, diffusion measures, and tract profiles of six patients with optic nerve atrophy. (**A**) A plot indicating the average temporal-superior-nasal-inferior-temporal RNFL thickness of optic nerve head, measured at a circle with a diameter of 3.4 mm centered on the disc by optical coherence tomography. The RNFL thickness of the atrophic eyes (solid line) was significantly reduced compared to that of contralateral non-affected eyes (dashed line). (**B**) Optic nerve pathway mapping by the average fractional anisotropy and radial diffusivity ($\upmu$m^2^/s) values for six atrophic optic nerves (ONs) and six non-affected ONs are presented in the left and right, respectively. (**C**) Average tract profiles across the ONs of six atrophic (red) eyes and six non-affected (gray) eyes plotted by mean lines with standard deviation and each node marked with dotted lines. Five nodes nearest the optic nerve head were excluded from the analysis and are shaded white in the graph.

DTI was preprocessed for denoising and corrected for eddy current and motion artifacts using MRtrix3 software (http://www.mrtrix.org) based on published methods^15–19^. The susceptibility-induced off-resonance field and subject head motion was estimated using two pairs of b0 images with distortions in opposite directions. One corrected b0 image was generated, and then this estimated field was applied to correct the entire diffusion data. This method is based on registering the individual volumes of the diffusion data set to a model-free prediction to account for unique eddy current distortions present in each volume and any subject movement between the acquisition of successive volumes^20^. Subsequent processing was performed using the mrDiffusion implemented in Vistasoft (https://github.com/vistalab/vistasoft). The corrected diffusion data, as a set of 49 images, were co-registered to the anterior commissure—posterior commissure aligned T1 image using a rigid-body mutual information algorithm. Diffusion-weighting gradient directions were reoriented by applying the same transformation used on the diffusion-weighted images and resampled to 2 mm isotropic voxels using trilinear interpolation. Finally, the tensors were estimated to compute FA, MD, RD, and AD using a least-squares algorithm with bootstrapping for 500 times^21^. Overview of the DTI analysis and details of diffusion measures for ONs are illustrated in Fig. 1. Plotting tensor model and fiber tract on MRI was done using FSLeyes (v0.34.2) https://fsl.fmrib.ox.ac.uk/fsl/fslwiki/FSLeyes) and Matlab Brain Anatomy (MBA, https://github.com/francopestilli/mba). The main diffusion properties for FA and RD in our analysis are visualized in Fig. 2B.

### ON tractography

The region of interest (ROI) for each intraorbital ON was manually identified. ROI placement was based on the co-registered T1-weighted image with b0 image. A 3-mm sphere was used slightly posterior to the globe of the eye, while another was placed in the orbital apex at the center of the annulus of Zinn, where the extraocular muscles were confluent (Fig. 1D)^22^. The ON fiber tracking was performed using ConTrack to estimate the most likely pathway between a pair of ROIs (https://github.com/vistalab/contrack). This tool was used to identify visual pathways in previous studies^23,24^. A set of 1000 candidate pathways for ON was generated, and the top 10% of the most likely fibers were selected by the ConTrack scoring algorithm. Additional fiber cleaning was performed using the automated fiber quantification (AFQ) toolkit (https://github.com/jyeatman/AFQ), following the prescriptions of previous studies^12,25,26^. Outlying fibers were removed when their distance was more than 2.6 standard deviations away from the pathway’s fiber core or more than three standard deviations longer than the average fiber length. To overcome some flaws regarding the low resolution of diffusion MRI voxels and partial volume effect caused by too small structure for ON, the final ON pathway was restricted to a central fiber passing inside the ROI by averaging the pathway’s core fibers. It was then normalized as 20 equidistant nodes along the ON pathway length. Diffusion measures such as FA, MD, RD, and AD were taken with 20 values assigned to nodes along the length of the pathway. We excluded the five nodes nearest to the ONH and averaged ON diffusion measures from the posterior 75% (15 nodes) of intraorbital ON to reduce the effect of eye movement during MRI acquisition.

### Statistical analysis

Diffusion measures and RNFL thickness of atrophic and non-affected ONs in each patient with unilateral OA were analyzed. In the control group, there were no significant differences in diffusion measures and RNFL thickness between both ONs (FA, *P* = 0.948; MD*, P* = 0.293; AD, *P* = 0.237; RD, *P* = 0.393, RNFL thickness, *P* = 0.741). Therefore, the averaged measurements of both ONs in each subject were used for statistical analysis. To analyze the patients’ demographic and clinical characteristics of the included ONs, the Mann–Whitney *U* test was used for non-parametric continuous variables, and the χ^2^ test was used for categorical variables. Statistical significance was reported at *P* < 0.05.

A linear mixed-effects (LME) model was used to examine the association between the averaged diffusion measures and RNFL thickness as a fixed effect and subject as a random effect. These models followed the general structure: *Y*_*FA/MD/AD/RD*_ ~ *RNFL* + *(1|Subject)*. To compare group differences in diffusion measures and RNFL thickness, this model factored “atrophic” versus “control” or “non-affected”, and the group term was included as a fixed effect and subject as a random effect following as *Y*_*FA/MD/AD/RD*_ ~ *Group* + (*1|Subject*). The reported P-values are from the analysis of variances of the fixed effects. All statistical analyses and plots were conducted using the MATLAB (R2017b) statistical toolbox (MathWorks, Natick, MA, USA).

## Results

### Demographics and clinical characteristics

Participants’ demographics and the clinical characteristics of the eyes are summarized in Table 1. Age and sex distributions were similar between the OA and control groups (*P* = 0.083 and *P* = 0.232, respectively). The etiology of five of the six unilateral OAs was trauma. One patient developed toxic optic neuropathy due to carbon monoxide poisoning. The time from the event/injury to examination ranged from 5 to 59 months. RNFL thickness of ONH was significantly lower in atrophic eyes than in non-affected and control eyes (*P* < 0.001, both). Figure 2A presents a comparison of the average RNFL thickness of atrophic and non-affected ONs.

**Table 1:** Demographics and clinical characteristics of patients with optic atrophy (OA group) and control subjects (control group).

	Age (years)	Sex	Diagnosis [time from onset]	RNFL ($\mu m$)	
				Affected	Non-affected
**Cases (OA group)**					
Case 1	54	M	Traumatic optic neuropathy [7 months]	51	113
Case 2	45	F	Traumatic optic neuropathy [5 months]	60	108
Case 3	60	F	Toxic optic neuropathy [16 months]	37	109
Case 4	60	M	Traumatic optic neuropathy [6 months]	62	105
Case 5	60	M	Traumatic optic neuropathy [59 months]	48	103
Case 6	66	M	Traumatic optic neuropathy [30 months]	41	121
**Groups**					
OA group (N = 6)	57.50 ± 7.20	33%*		49.83 ± 9.99^a^	109.83 ± 6.46^b^
Control group (N = 11)	50.55 ± 8.03	64%*		109.64 ± 4.99^c^	
*P*-value	0.083^§^	0.232^†^		< 0.001^††^	

Continuous variables in each group are presented as the mean ± standard deviation.

*N* number of subjects, *OA* optic atrophy, *RNFL* total retinal nerve fiber layer thickness, *F* female, *M* male.

*Female ratio percentage.

^**†**^Chi-square test.

^††^Mann–Whitney U test (a *vs.* b, a *vs.* c).

^§^Mann–Whitney U test.

^a^Mean of RNFL thickness in affected eyes. ^ b^Mean of RNFL thickness in non-affected eyes. ^c^Mean of averaged RNFL thickness in both eyes.

### Group difference in diffusion measures

**Figure 3:**
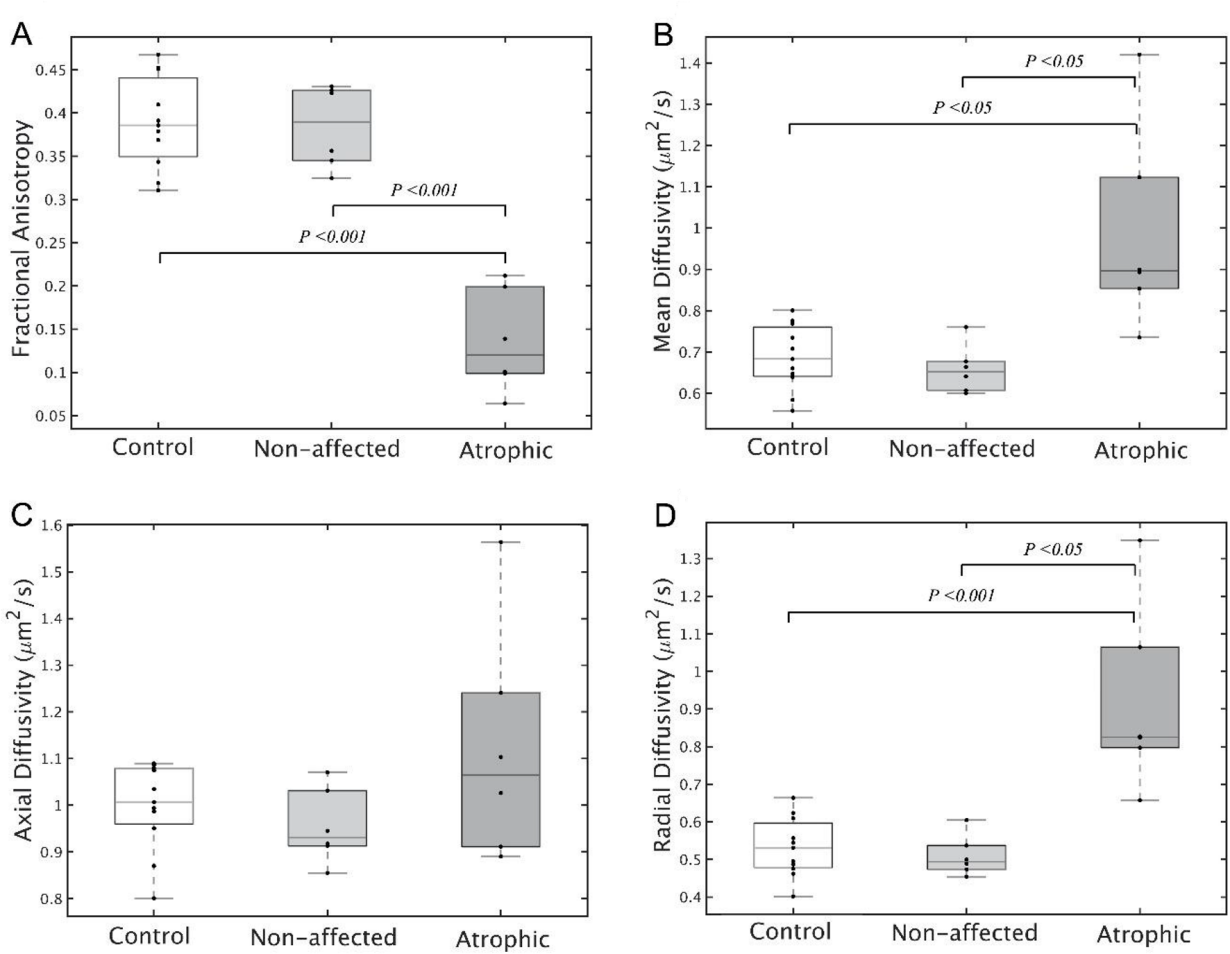
Comparison of diffusion measures of atrophic optic nerves (ONs) to those of non-affected ONs and control ONs. Comparison of averaged optic nerve fractional anisotropy (FA) (**A**), mean diffusivity (MD) (**B**), axial diffusivity (AD) (**C**), radial diffusivity (RD) (**D**) in controls (n = 11) and patients (n = 6) with the light gray boxplot for non-affected eyes and dark gray boxplot for eyes with atrophic optic nerves. On each box, the central mark is the median, the edges of the box are the 25th and 75th percentiles, and data points are plotted individually. Significant differences are denoted by the marked line with the p-value.

Group differences in diffusion measures of the ONs are shown in Fig. 3. FA of atrophic ON was significantly lower than that of non-affected and control ONs (atrophic [A], 0.136 ± 0.059; non-affected [N], 0.384 ± 0.048; control [C], 0.389 ± 0.053; A vs N, *P* < 0.001; A vs C, *P* < 0.001). Also, MD and RD of atrophic ONs were significantly higher than those of non-affected and control ONs (MD, A, 0.988 ± 0.247; N, 0.658 ± 0.058; C, 0.687 ± 0.079; A vs N, *P* = 0.010; A vs C, *P* = 0.002; RD, A, 0.920 ± 0.247; N, 0.510 ± 0.054; C, 0.532 ± 0.078; A vs N, *P* = 0.003; A vs C, *P* < 0.001). AD did not show significant differences between the groups (A, 1.123 ± 0.252; N, 0.955 ± 0.080; C, 0.998 ± 0.094; A vs N, *P* = 0.152; A vs C, *P* = 0.155). Figure 2B,C visualizes the significance differences in average FA and RD between atrophic and non-affected ONs. Figure 2C shows the average tract profiles across the ONs.

### Association between diffusion measures and RNFL thickness of ONH

**Figure 4:**
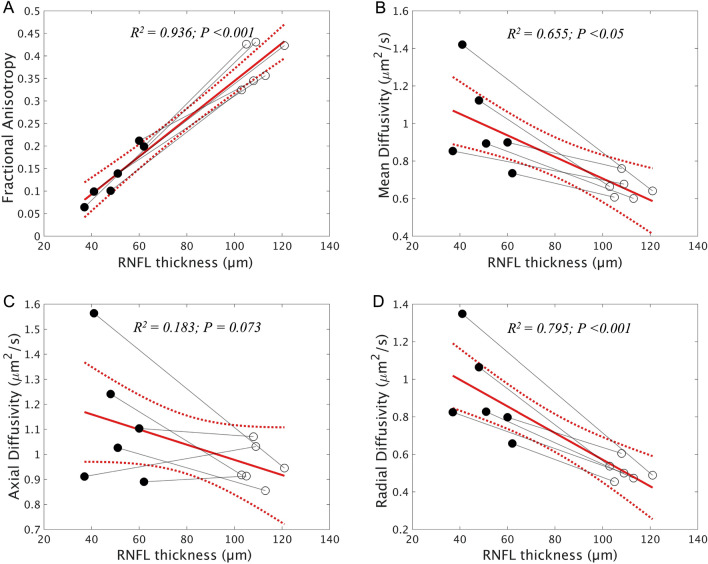
Correlation of diffusion measures and retinal nerve fiber layer (RNFL) thickness of the optic nerve head of atrophic ONs in patients with unilateral optic atrophy. Correlation between average RNFL thickness and average fractional anisotropy (**A**), mean diffusivity (**B**), axial diffusivity (**C**), and radial diffusivity (**D**). Correlations within patients are indicated by each solid gray line, with closed points marking eyes with atrophic optic nerves and open points marking non-affected eyes. A least-squares regression estimate is indicated by the solid line with red color and its 95% confidence bounds as the dashed line.

Diffusion measures of the intraorbital ON showed varying degrees of correlation with the average RNFL thickness of ONH in atrophic ONs (Fig. 4). The strongest positive correlation was found between RNFL thickness and FA (*R*^2^ = 0.936. *P* < 0.001), followed by RD and MD (RD, *R*^2^ = 0.795, *P* < 0.001; MD, *R*^2^ = 0.655, *P* = 0.001), while the AD showed no significant correlation with the RNFL thickness $($*R*^2^ = 0.183, *P* = 0.073).

## Discussion

The present study found distinct changes in diffusion measures (FA, MD, and RD) in ON of eyes with OA compared to normal eyes. These measures also showed a significant correlation with the RNFL thickness of ONH. Our methodology enabled a more accurate evaluation of the neural properties of ONs in that we applied the most recent technique to correct artifacts and extracted the central line of the ON on the dMRI to reduce the partial volume effect. To the best of our knowledge, this was the first study to quantitatively measure the neural properties of ONs using DTI in OA and demonstrate the clinical relevance of DTI measurements.

DTI studies of ophthalmic disease in which ON is affected such as glaucoma, optic neuritis, and other optic neuropathies have revealed reduced FA, where smaller values indicate multiple intersections, degeneration, or demyelination in the visual pathway, and increased MD, where larger values indicate a more diffuse (“weaker”) pathway and smaller values indicate a denser (“strong”) pathway^11–13,27,28^. Li et al*.* investigated DTI measures of patients with traumatic optic neuropathy (TON), where the damage occurred three months before the examination in almost all patients, and also showed decreased FA and increased MD^29^. Our findings of lower FA and higher MD in ONs with OA where injury to ON occurred at least 5 months before the MRI scan are in agreement with previous studies. In general, larger FA and smaller MD values are known to be associated with compact, uniformly oriented bundles of axons^12^. Therefore, in ON with OA, smaller FA and larger MD values can be expected. ON consists of bundles of axons arranged in parallel, with membranes and myelin sheaths being the main barriers to the diffusion of water molecules. In OA, the degeneration of axon and myelin sheaths and the replacement of neurons with glial cells and connective tissues may reduce the anisotropy of the diffusion process^30^. The lower tissue density and/or less impedance may also induce greater diffusivity.

The directional diffusivities, AD and RD, were also analyzed. In atrophic ON, RD was significantly higher and significantly correlated with RNFL thickness, whereas this significance was not found in AD. AD has been known to be more indicative of axonal degeneration, while RD is more indicative of changes in white matter myelination in the ON diseases^9,10^. AD has been reported to show an initial decrease in acute optic neuritis. However, a significant increase during the remote stage in patients with optic neuritis followed longitudinally and therefore is suggested to be a marker of *acute* axon injury^11,31^. It was suggested that organelle and protein accumulation resulting from the failure of ion channels and transport mechanisms in axons in the setting of the acute demyelinating disease might lead to increased intra-axonal viscosity, contributing to compromised axonal transport, which in turn result in a decrease in AD^11^. They also suggested that, after the myelin debris is cleared up, the axons reduce their contribution to diffusivity by constituting a smaller proportion of the tissue, representing the normalization of AD in the remote stage^11^. This hypothesis may explain the non-significant difference in AD in ON with OA in our study. As OA is the end stage of axonal damage, acute alterations in axonal transport should not be demonstrated, and therefore there should not be a significant difference in AD. A previous DTI study on TON showed decreased AD in the injured ON, but the time from the injury to the DTI scan was mostly less than 95 days^29^. Meanwhile, RD has been reported to show no significant alteration at the acute stage of optic neuritis but shows a continuous increase in the remote stage^11,32^. Increased RD also has been reported in ischemic optic neuropathy^14^ and TON^29^, in which a progressively increasing trend of RD was reported. Our finding of a significant increase in RD in ONs with OA is consistent with previous reports. Chronic alterations in the ONs due to loss of myelin and axons can result in increased diffusion perpendicular to the parallel axons^33,34^.

RNFL thickness of ONH measured using OCT is the most useful measurement in optic nerve diseases in clinical practice. The present study showed a significant correlation between diffusion measures and RNFL thickness of ONH in ONs with OA. FA showed the strongest correlation with RNFL thickness, and RD and MD also showed a significant negative correlation. The correlation between diffusion measures and RNFL thickness was reported in several studies with optic neuritis and glaucoma patients^32,35,36^. The correlation between MD and FA and RNFL thickness in patients with primary open-angle glaucoma at different stages has been reported^36^, and RD was reported to be strongly correlated with RNFL thickness (negative correlation) in ONs with chronic optic neuritis^32^. Wang et al*.* found that the same four DTI parameters (FA, MD, RD, and AD) were correlated with RNFL thickness in ONs with primary closed-angle glaucoma, whereas only FA and RD were significantly correlated with the glaucoma stage, suggesting that FA and RD values could be more reliable in assessing ON damage^35^. The different results in AD may come from the study population where they included all stages of glaucoma (early, moderate, and severe defect), and OA likely corresponds to the late or severe stage of glaucoma. In this stage, the AD value of ON may not show a significant difference. Therefore, it did not show a significant correlation with RNFL thickness. Additionally, a significant correlation between decreased FA and increased MD and RD with peripapillary RNFL thickness has also been reported in patients with TON^29^. Overall, these results suggest a strong link between RNFL thickness, one of the most important instrumental measurements of ON diseases, and diffusion measures that indicate the structural integrity of the ON axons.

Miller and Allen successfully showed tractography-based studies for the entire ON structure in glaucoma patients and amblyopia^12,26^ using advanced DTI protocol and recently suggested an artifact correction method^20^. Previously, most studies have relied on manual ON segmentation or ROI-based analyses restricted by small portions of the ON^13,27^. Following the tractography-based studies mentioned above, we acquired a set of anteroposterior and posteroanterior phase-encoded b0 images and applied the artifact correction method to recover from imaging distortions caused by the adjacent nasal cavities and improve signal-to-ratio. However, it remained challenging to accurately extract ON fibers with visible OA in diffusion data because the intensities of the optic nerve boundaries were not clearly distinguished from the surrounding cerebrospinal fluid. Indeed, in the fibers of ON with OA, diffusion measures were not estimated as densely as in normal agglomerate fiber bundles. We successfully extended the tractography-based approach by generating a central track line that represents core ON fibers and taking obvious diffusion measures by sub-sampling on a voxel that passes the center of the ON structure along the entire ON length. It would be advantageous as we could avoid partial volume effects from surrounding tissues which are inevitable in a voxel-based (2 × 2 × 2 mm^3^) analysis of diffusion data because of the relatively small size of the ON structure with a diameter of about 4 mm.

Furthermore, we analyzed only the intraorbital part of ON to remove other ambiguous effects that can be resulted from adopting the tractography technique for the entire ON, from ONH to optic chiasm, as typically analyzed in previous studies^12,26,37^. ON is composed of three anatomical segments: intraorbital, intracanalicular, and intracranial segments. Haykal et al. recently showed distinct changes in the intraorbital ON and the intracanalicular ON in glaucoma patients. However, when we performed the tractography in the intracanalicular and intracranial parts of ON, we found that the profiling of the fiber showed fluctuation and did not show a uniform structure along the path even in normal subjects not like in the intraorbital ON. Their diffusion measures showed a wide distribution from the mean value with a large standard deviation. The intracanalicular and intraorbital segments are more likely to be affected by magnetic susceptibility artifacts because those are in closer proximity to the magnetic field distortions from paranasal sinus and skull base compared to the intraorbital segment showing lower effect than other segments^37,38^.

This study has several limitations. First, the sample size of the unilateral OA group was relatively small; therefore, group matching may not be sufficient to reflect changes in RNFL thickness with age and sex^39^. However, it is a strength of this study that we included patients with unilateral OA to investigate the inter-eye difference in the same patient to provide reliable characteristic features of atrophic ONs. Second, the resolution of dMRI was relatively low with respect to the small structure for ON because we acquired a whole-brain scan to observe other visual pathways additionally. The resolution can be increased when using a regional field of view by optimizing a dMRI protocol and a dedicated surface coil in future studies. Third, although DTI measures have been widely used in many studies, they are not tissue-specific parameters as they inherently represent properties based on a single compartment. The tensor model cannot distinguish different tissue types within a voxel since it depends on a major principal direction. This could lead to difficulties in interpreting DTI results related to the scalar. Therefore, our findings were interpreted with caution by considering the patients’ clinical history. Further studies are needed to investigate ON structures more comprehensively by adopting high-order models which overcome the limitations of the current DTI technique.

In conclusion, this study was the first to elucidate the clinical significance of diffusion measures in patients with OA using the most recent technique to reduce measurement errors. In ON with OA, distinct changes were found in diffusion measures: lower FA and higher MD and RD. These diffusion parameters may also serve as quantifiable assessments of OA, corresponding to the RNFL thickness of ONH. Our findings may throw light on the understanding of the ultrastructural changes in ON axons in ON diseases and help extend the clinical application of DTI in ophthalmic diseases.

## Data Availability

The data that support the findings of this study are available from the corresponding author upon reasonable request.
